# Virtual Care Expansion and Physician Follow-Up Among Youth With First Mental Health Contact Through a Hospital: Population-Based Repeated Cross-Sectional Study

**DOI:** 10.2196/94389

**Published:** 2026-09-09

**Authors:** Erica Wennberg, Emily Hamovitch, Aditi Patrikar, Peter C Austin, Paul Kurdyak, Katherine E Nelson, Amreen Babujee, Astrid Guttmann

**Affiliations:** 1 Institute of Health Policy, Management and Evaluation Dalla Lana School of Public Health University of Toronto Toronto, ON Canada; 2 Temerty Faculty of Medicine University of Toronto Toronto, ON Canada; 3 Child Health Evaluative Sciences SickKids Research Institute Toronto, ON Canada; 4 ICES Toronto, ON Canada; 5 Edwin S.H. Leong Centre for Healthy Children Toronto, ON Canada; 6 Centre for Addiction and Mental Health Toronto, ON Canada; 7 The Hospital for Sick Children Toronto, ON Canada

**Keywords:** virtual care, follow-up, first contact, access, mental health services

## Abstract

**Background:**

Timely follow-up is crucial for youth whose first mental health contact is through a hospital; virtual care could facilitate this.

**Objective:**

This study aimed to examine the association between the significant expansion of virtual care in Ontario, Canada, and follow-up mental health care rates for these youths.

**Methods:**

We conducted a population-based repeated cross-sectional study using linked health administrative databases. We identified first-contact emergency department (ED) visits or hospital admissions for self-harm, mood disorders, or psychosis from March 1, 2009, to February 29, 2024, among Ontario youth (aged 10-24 years). Virtual care was expanded in March 2020; we examined 2 expansion stages (temporary and permanent). Autoregressive integrated moving average analyses estimated level and slope changes (vs before virtual care) in age- and sex-standardized monthly rates of 7-day mental health follow-up. Subgroup analyses examined rurality and socioeconomic status.

**Results:**

We identified 42,208 first-contact ED visits and 17,701 first-contact hospital admissions. Pre–virtual care mean follow-up rates were 17.64 (SD 2.97) per 100 first-contact ED visits and 21.90 (SD 5.05) per 100 first-contact hospital admissions. Temporary expansion was associated with immediate increases in follow-up that declined (level changes: 4.81, 95% CI 2.82-6.81 for ED visits and 7.51, 95% CI 4.41-10.60 for hospital admissions; slope changes: −0.31, 95% CI −0.42 to −0.21 for ED visits and −0.32, 95% CI −0.48 to −0.15 for hospital admissions). Permanent expansion was associated only with an immediate decrease in ED visit follow-up (level change: −3.81, 95% CI −6.70 to −0.92) that was significant in rural (−1.06, 95% CI −1.93 to −0.184) but not urban (0.44, 95% CI −0.179 to 1.06) areas; results were otherwise similar between subgroups.

**Conclusions:**

Virtual care was not associated with sustained changes or equity improvements in mental health follow-up for high-acuity youth, representing a missed opportunity to extend physician reach and address barriers. Further research and quality improvement are needed.

## Introduction

Timely follow-up care is crucial for youth with acute mental health care needs. One group that may be particularly at risk of poor follow-up are youth whose first mental health care contact occurs through an emergency department (ED) visit or hospital admission [1]. These visits and admissions constitute a high proportion of acute mental health presentations among US Medicaid beneficiaries and in Canada (32%-54%) [1,2]. Due to the absence of prior care for mental health concerns and a lower likelihood of having a regular primary care provider [1], they may require more complex follow-up arrangements. Of all first-contact youth, those presenting for self-harm, mood disorders, and psychosis are likely to be at highest risk of adverse outcomes after discharge and require close follow-up. Relative to other mental health presentations, self-harm, mood disorders, and psychosis are associated with higher risk of return ED visits, mental health admissions and readmissions, and suicide [3-5].

Follow-up within 7 days after discharge has been shown to reduce risk of suicide following psychiatric hospitalization [6,7] and is recommended by US and Canadian quality standards for psychiatric acute care [8-11]. Quality standards in Ontario, Canada, specifically recommend follow-up with a trained mental health physician within 7 days following hospitalization for schizophrenia and follow-up with a psychiatrist or primary care clinician within 7 days following an acute care visit for depression [9,10]. However, youth may face substantial barriers to mental health follow-up, including limited availability of services, lack of continuity of care, stigma and negative experiences or beliefs around seeking help, negative experience with involuntary psychiatric hospitalization, and access barriers (eg, financial cost) [12-18]. Barriers to follow-up may be accentuated among first-contact youth. On the basis of estimates from Medicaid databases, 7-day follow-up for self-harm, mood disorders, and psychosis ranges from 30% to 60% depending on admission status, with substantially lower follow-up among youth with first-contact ED visits (17%) [2,19]. In Canada, 7-day follow-up rates are lower overall (approximately 30% for youth hospitalized for self-harm, a mood disorder, or psychosis in Ontario) [20]; follow-up rates among first-contact youth are, to our knowledge, currently unknown.

One promising opportunity for improving access to follow-up care is virtual care. Use of virtual mental health care became widespread following the onset of the COVID-19 pandemic. In Ontario, the proportion of child and adolescent outpatient mental health care delivered virtually increased from less than 5% to more than 90% in the first 2 months of the pandemic [21]. Similar patterns were observed in other jurisdictions; in US cohorts, use increased from less than 1% to between 46% and 59% in the early months of the pandemic [22,23]. Blended virtual and in-person postdischarge services are also emerging for youth [24,25]. With the widespread adoption of virtual care came the potential for increased and more equitable access, notably for individuals with existing barriers to accessing in-person care (eg, virtual care could resolve barriers based on distance from hospitals or clinics, lack of transportation, or difficulty taking time off school or work). Virtual care could also enable physicians to extend their reach beyond their usual patient population. While qualitative studies have described access improvements with virtual care for subsets of youth (eg, those in rural areas), these reports have been conflicting [26,27]. Youth and mental health care professionals have also described conflicting impacts of virtual care on ease of use, privacy, and engagement [26,28-30]. To our knowledge, there have been no population-level quantitative studies in this area. It is still unknown whether virtual care resulted in improved access to mental health care for first-contact youth at a population level and largely unknown whether it has done so for youth with mental health care needs generally.

To fill this gap, we leveraged population-based data from Ontario, Canada’s most populous province, and the widespread expansion of virtual care across its universal health care system. Virtual care services in Ontario’s universal health care system comprise telephone or video visits with a general practitioner or specialist, with video visits offered through any verified virtual visit solution [31]; choice of platform is at the discretion of the physician or their organization. Ontario expanded physician remuneration for telephone and video visits in response to the COVID-19 pandemic through the introduction of new virtual billing codes in March 2020, leading to widespread uptake of virtual care; these codes were designated as temporary and were replaced with permanent codes in December 2022 [32]. Updates were made between these 2 billing code periods, most importantly reduced compensation for virtual visits without an existing patient-physician relationship compared to those with a preexisting relationship. The different compensation structures were meant to support continuity of care, disincentivizing provision of virtual visits in the absence of a preexisting patient-physician relationship. Another central difference was the pandemic vs postpandemic context of the 2 periods. Our objective was to examine the association between the expansion of virtual care through temporary and permanent billing codes in Ontario, Canada, and rates of follow-up mental health care among youth whose first mental health contact for self-harm, a mood disorder, or psychosis was through an ED visit or hospital admission. To investigate potential improvements in equity, we also explored differences by rurality and socioeconomic status. We hypothesized that the expansion of virtual care was associated with both short- and long-term improvements in access to and equity in follow-up care.

## Methods

### Study Design

We conducted a population-based repeated cross-sectional study of Ontario youth and young adults (aged 10-24 years) discharged home between March 1, 2009, and February 29, 2024, following an ED visit or hospital admission as a first mental health care contact for self-harm, a mood disorder, or psychosis. First-contact ED visits were defined as ED visits preceded by no outpatient or inpatient mental health service use in the prior 2 years [1] and not resulting in hospital admission; those resulting in hospital admissions were defined as first-contact hospital admissions. Diagnostic categories were mutually exclusive (self-harm, mood disorder without self-harm, and psychosis without self-harm). Detailed definitions for the population, demographic characteristics, and all other variables can be found in Table S1 in Multimedia Appendix 1. We chose to focus on self-harm, mood disorders, and psychosis due to their clinical importance and to maximize homogeneity in follow-up requirement; all have a high-risk postdischarge period and high indication for follow-up [3-5]. All individuals included in the study were eligible for Ontario’s universal health care system. Nonresidents of Ontario were excluded (Figure S1 in Multimedia Appendix 1). Study members were included in monthly cross-sectional cohorts based on their date of discharge and followed until their outcome date (the date of their first mental health follow-up visit, defined further in the Outcome section below and in Table S1 in Multimedia Appendix 1), death, or 30 days after discharge. The maximum follow-up date was March 30, 2024. First-contact ED visits and hospital admissions were evaluated separately in all analyses. Due to the 2-year lookback for first-contact visits, it was possible for a unique participant to have more than one first-contact visit across the 15-year study period; these were considered independent. The gap between first-contact visits from the same individual was at minimum 2 years. A minority of included visits were from an individual already appearing in the cohort (approximately 5%). We followed relevant reporting guidelines [33,34].

### Data Sources

We used Ontario health administrative and demographic databases linked at the person level using unique encoded identifiers and analyzed at ICES (formerly the Institute for Clinical Evaluative Sciences). ICES is an independent, nonprofit research institute whose legal status under Ontario’s health information privacy law allows it to collect and analyze health care and demographic data without individual patient consent for health system evaluation and improvement. We used ambulatory care, hospital discharge, and mental health inpatient databases to identify ED visits and hospital admissions for self-harm, mood disorders, and psychosis, as well as prior mental health service use. Community health center and provincial physician billing record databases were used to identify mental health follow-up and prior mental health service use. Demographic databases (eg, the provincial health insurance registry and federal immigration data) were used to determine additional demographic characteristics. Details on databases can be found in Table S2 in Multimedia Appendix 1.

### Exposure and Comparator Periods

Our exposure periods were the expansion of virtual care in Ontario’s universal health care system through temporary (March 14, 2020, to November 30, 2022) and permanent (December 1, 2022, to February 29, 2024) billing codes [32,35]. We applied a washout period from March 2020 to May 2020 to address initial variability in use of temporary billing codes and exclude the concurrent initial months of COVID-19 pandemic restrictions. Our comparator was the period preceding virtual care (March 1, 2009, to February 28, 2020). While remuneration for virtual care existed in Ontario during this period, its use was rare (<3% of adult visits and <1% of outpatient pediatric mental health visits) [21,36].

### Outcome

The primary outcome was the age- and sex-standardized monthly rate of 7-day follow-up mental health care; 14- and 30-day follow-ups were secondary outcomes. Follow-up mental health care was defined as a virtual or in-person outpatient mental health visit with a general practitioner, pediatrician, or psychiatrist identified using a validated algorithm modified for pediatric care [37]. We focused on physician follow-up due to high potential for youth to be starting medications after a first-contact visit for self-harm, psychosis, or a mood disorder and guidelines recommending physician follow-up for these conditions; nonphysician services are also not captured in ICES databases. For ED visits, the outcome window began on the first day following discharge as billings for outpatient services on the discharge date cannot be distinguished from some consultations in the ED. For hospital admissions, the outcome window was inclusive of the hospital discharge date (ie, postdischarge day 0). For each observation, only the first follow-up visit was considered. Monthly follow-up rates were calculated as the number of first-contact visits with follow-up in each month per 100 first-contact ED visits or hospital admissions. Rates were directly standardized by age and sex using the observed age (10-17 and 18-24 years) and sex (male and female) distributions in 2023 as the reference population. For subgroup analyses, subgroup-specific 2023 distributions were used.

### Statistical Analysis

We performed interrupted time-series analyses using autoregressive integrated moving average (ARIMA) models [38] to estimate changes (vs before virtual care) in age- and sex-standardized monthly follow-up rates. We chose ARIMA as we expected autocorrelation and seasonality in the outcome. ARIMA models estimated level and slope changes during each of the exposure periods compared to the pre–virtual care period and produced forecasted rates. Level changes represented the immediate change in follow-up rates in the first month of each virtual care period compared to before virtual care, whereas slope changes represented the change in the rate of change of follow-up rates in each virtual care period compared to before virtual care. Analyses were conducted using R (version 4.0.2; R Foundation for Statistical Computing). For each model, differencing orders were selected manually, and optimal *p* and *q* values were determined using the *auto.arima* function [39]. Models with a first-order difference, a seasonal difference, and no differencing were fit using *auto.arima*, and the model with the lowest root mean squared error (used in place of the Akaike information criterion or Bayesian information criterion as they assume equal observations across datasets) was chosen; residual plots were also examined. This approach was used as traditional approaches (data visualization, stationarity, and seasonality tests) produced conflicting results on the optimal specification for each model. Results from ARIMA models with alternate differencing orders are presented for our primary analyses.

To test differences by important measures of equity, we conducted subgroup analyses by residence in rural vs urban areas and areas with lower vs higher material resources. “Rural” was defined using Rurality Index of Ontario scores of 40 or higher [40]; missing values were grouped with “rural.” Material resource quintiles were defined using the Ontario Marginalization Index [41], which is based on Canadian census data. Quintiles were assigned by census dissemination area, the smallest geographic area in the Canadian census, with an average population size of 400 to 700 individuals. The material resources dimension provides a holistic, neighborhood-level measure of socioeconomic status; it includes indicators such as proportion with low income, unemployed, and living in dwellings in need of major repair. Areas with lower vs higher material resources were defined as quintiles 4 and 5 vs 1, 2, and 3; missing values were grouped with lower–material resource areas. We conducted two sensitivity analyses: (1) changing the primary outcome to age- and sex-standardized monthly rates of 7-day follow-up for any indication (to capture potential use of not mental health–specific diagnostic coding by physicians), defined as any outpatient visit, including planned ED visits; and (2) changing our primary analysis model to segmented linear regression (to test the robustness of our findings to the use of a different model type). Baseline tables examined participant characteristics within each period using means, medians, and proportions, with differences between periods assessed using standardized differences due to our large sample size (≥0.1 considered meaningful). Tables of follow-up characteristics within each period were created using the same methods. To accompany our analyses, we also generated other descriptive summaries (box plots and bar charts) of follow-up rates and characteristics in each period, as well as graphs of the monthly rates of first-contact visits over time. Table S1 in Multimedia Appendix 1 provides detailed definitions of all variables.

### Ethical Considerations

ICES is a prescribed entity under Ontario’s Personal Health Information Protection Act (PHIPA). Section 45 of PHIPA authorizes ICES to collect personal health information, without consent, for the purpose of analysis or compiling statistical information with respect to the management of, evaluation or monitoring of, the allocation of resources to, or planning for all or part of the health system. Projects that use data collected by ICES under section 45 of PHIPA, and use no other data, are exempt from research ethics board review. The use of the data in this project is authorized under section 45 and approved by ICES’ Privacy and Legal Office. Secure access to these data is governed by policies and procedures that are approved by the Information and Privacy Commissioner of Ontario [42,43].

## Results

### First-Contact ED Visits

#### Visit Characteristics

This study included 42,208 first-contact ED visits for self-harm, a mood disorder, or psychosis: 33,076 (78.4%) in the pre–virtual care period (approximately 250 per month), 6269 (14.9%) in the temporary billing code period (approximately 209 per month), and 2863 (6.8%) in the permanent billing code period (approximately 191 per month; Table 1). First-contact ED visits rose from 2009 to 2017, then declined (Figure S2 in Multimedia Appendix 1). Differences in visit characteristics between the temporary and permanent billing code periods compared to the pre–virtual care period were observed in age (increasingly younger in each period), time of residence in Ontario (shorter in the permanent billing code period), rurality (more rural residents in the permanent billing code period), and primary care attachment (lower in each period). Sex, diagnosis, and material resource distributions were similar across periods.

**Table 1:** Characteristics of first-contact emergency department visits by period^a^.

Characteristics		Pre–virtual care period (March 1, 2009-February 29, 2020; n=33,076; approximately 250 per mo)	TBC^b^ period (June 1, 2020-November 31, 2022; n=6269; approximately 209 per mo)	SMD^c^—TBC vs pre–virtual care period	PBC^d^ period (December 1, 2022-February 29, 2024; n=2863; approximately 191 per mo)	SMD—PBC vs pre–virtual care period
**Age (y)**						
	Mean (SD)	18.31 (3.43)	17.94 (3.76)	*0.10* ^e^	17.97 (3.74)	0.09
	Median (IQR)	18 (16-21)	18 (15-21)	*0.10*	18 (15-21)	0.09
**Age group (y), n (%)**						
	10-17	13,987 (42.3)	2971 (47.4)	*0.10*	1352 (47.2)	*0.10*
	18-24	19,089 (57.7)	3298 (52.6)	*0.10*	1511 (52.8)	*0.10*
**Sex, n (%)**						
	Female	18,124 (54.8)	3699 (59)	0.09	1671 (58.4)	0.07
	Male	14,952 (45.2)	2570 (41)	0.09	1192 (41.6)	0.07
**Discharge diagnosis, n (%)**						
	Any self-harm	11,671 (35.3)	2224 (35.5)	<0.01	1080 (37.7)	0.05
	Mood disorder without self-harm	19,734 (59.7)	3692 (58.9)	0.02	1600 (55.9)	0.08
	Psychosis without self-harm	1671 (5.1)	353 (5.6)	0.03	183 (6.4)	0.06
**Immigration status, n (%)**						
	Resettled refugee or protected person and other immigrants^f^	825 (2.5)	187 (3)	0.03	84 (2.9)	0.03
	Sponsored family immigrants	745 (2.3)	130 (2.1)	0.01	54 (1.9)	0.03
	Economic immigrants	1335 (4)	249 (4)	<0.01	101 (3.5)	0.03
	Long-term residents	30,171 (91.2)	5703 (91)	0.01	2624 (91.7)	0.02
**Time of residence in Ontario (y), n (%)**						
	<5	1560 (4.7)	392 (6.3)	0.07	274 (9.6)	*0.19*
	5-10	1310 (4)	242 (3.9)	0.01	122 (4.3)	0.02
	>10	30,206 (91.3)	5635 (89.9)	0.05	2467 (86.2)	*0.16*
**Material resource quintile, n (%)**						
	1 (highest)	4996 (15.1)	874 (13.9)	0.03	347 (12.1)	0.09
	2	5531 (16.7)	1120 (17.9)	0.03	443 (15.5)	0.03
	3	6052 (18.3)	1082 (17.3)	0.03	522 (18.2)	<0.01
	4	6509 (19.7)	1209 (19.3)	0.01	559 (19.5)	<0.01
	5 (lowest)	8805 (26.6)	1667 (26.6)	<0.01	778 (27.2)	0.01
	Missing	1183 (3.6)	317 (5.1)	0.07	214 (7.5)	*0.17*
**Rurality, n (%)**						
	Rural	5396 (16.3)	1084 (17.3)	0.03	575 (20.1)	*0.10*
	Urban	27,543 (83.3)	5165 (82.4)	0.02	2269 (79.3)	*0.10*
	Missing	137 (0.4)	20 (0.3)	0.02	19 (0.7)	0.03
**Primary care attachment^g^** **,** **n (%)**						
	Uncertainly attached, not receiving primary care services	3440 (10.4)	899 (14.3)	*0.12*	573 (20)	*0.27*
	Uncertainly attached, receiving primary care services	1398 (4.2)	267 (4.3)	<0.01	116 (4.1)	0.01
	Attached	27,892 (84.3)	5017 (80)	*0.11*	2133 (74.5)	*0.24*
	Missing	346 (1)	86 (1.4)	0.03	41 (1.4)	0.03

^a^Table S1 in Multimedia Appendix 1 provides additional information on variable definitions. Parts of this material are based on data and/or information compiled and provided by Immigration, Refugees, and Citizenship Canada current to September 2024.

^b^TBC: temporary billing code.

^c^SMD: standardized mean difference.

^d^PBC: permanent billing code.

^e^Italicized values indicate meaningful differences between periods (standardized mean difference≥0.1).

^f^“Protected person” refers to an individual granted refugee protection as a Convention refugee (individuals meeting the 1951 Geneva Convention definition of a refugee) or as a person in need of protection under Canadian law. Other immigrants are largely family members of refugees. They comprised <0.5% of the study population.

^g^“Primary care attachment” refers to affiliation of a patient with a regular primary care provider (enrolled with a primary care physician, clinic, or center or having continuity of care with a given primary care physician). Uncertain attachment refers to patients who are unaffiliated with a primary care provider. Table S1 in Multimedia Appendix 1 provides additional information.

#### Receipt of 7-Day Mental Health Follow-Up

**Figure 1:**
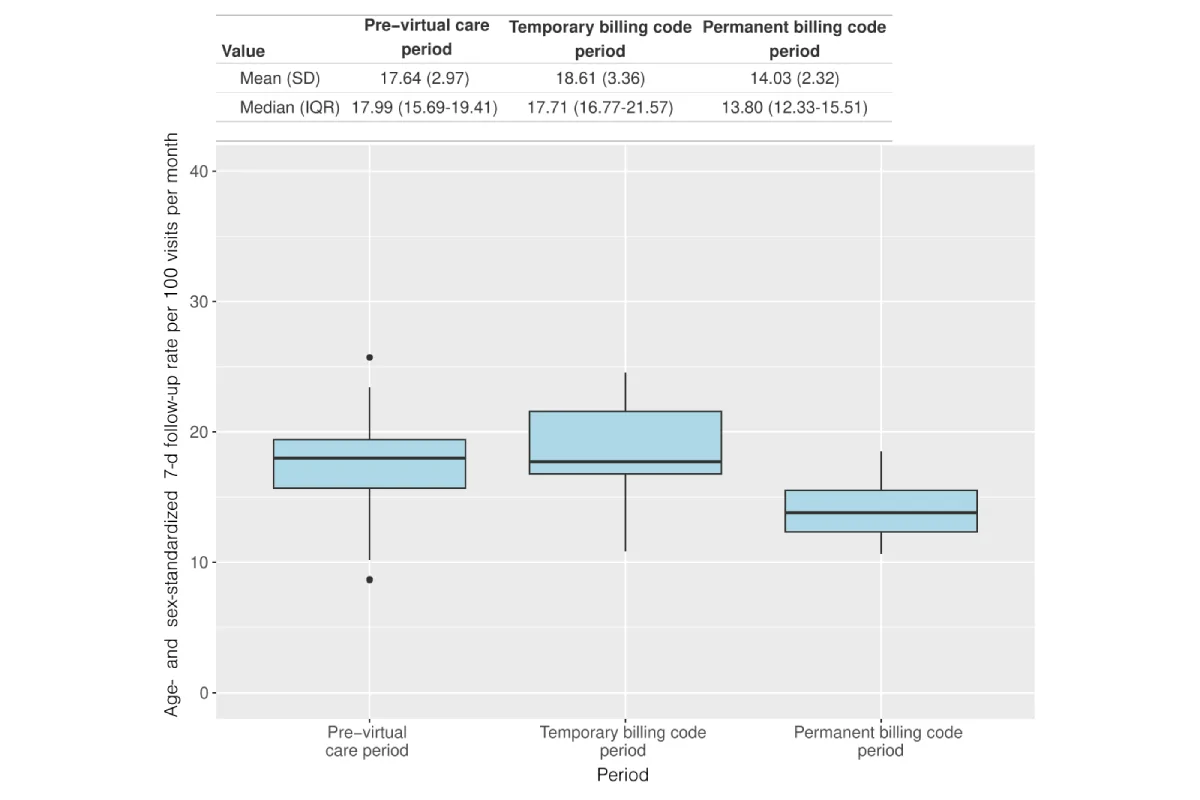
Distributions and average monthly rates of 7-day follow-up mental health care for first-contact emergency department visits by period.

**Figure 2:**
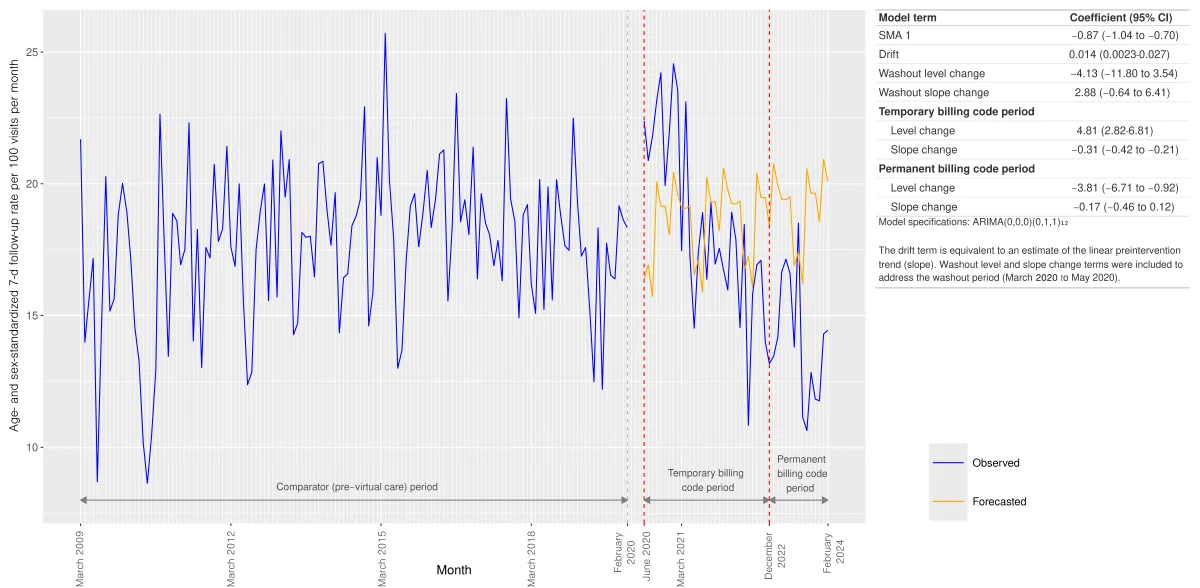
Time-series analysis results for monthly rates of 7-day mental health follow-up following first-contact emergency department visits for self-harm, mood disorders, or psychosis. ARIMA: autoregressive integrated moving average; SMA 1: first-order seasonal moving average.

In the pre–virtual care period, the mean standardized monthly rate of 7-day follow-up was 17.64 (SD 2.97) per 100 first-contact visits (Figure 1). Monthly follow-up rates rose slowly during this period at a rate of 0.014 follow-up visits per 100 per month (95% CI 0.0023-0.027). Temporary billing code introduction was associated with an immediate, significant increase in 7-day follow-up (level change, ie, immediate change in baseline rate vs the pre–virtual care period: 4.81, 95% CI 2.82-6.81), which then declined over time (slope change, ie, change in the monthly rate of change vs the pre–virtual care period: −0.31, 95% CI −0.42 to −0.21; Figure 2). Permanent billing code introduction was associated with an immediate, significant decrease in 7-day follow-up compared to the pre–virtual care period (level change: −3.81, 95% CI −6.70 to −0.92) and no significant change in the rate of change (slope change: −0.17, 95% CI −0.46 to 0.12). The mean standardized monthly rate of follow-up in the permanent billing code period was 14.02 (SD 2.32) per 100 first-contact visits. Sensitivity analyses and models with alternate differencing orders produced similar findings (Tables S3 and S4 and Figure S3 in Multimedia Appendix 1).

#### Receipt of 14- and 30-Day Mental Health Follow-Up

In the pre–virtual care period, mean standardized monthly rates of 14- and 30-day follow-up were 25.46 (SD 4.09; 1.44 times higher than 7-day rates) and 34.53 (SD 5.21; 1.96 times higher than 7-day rates) per 100 first-contact visits (Figure S4 in Multimedia Appendix 1). Changes in 14- and 30-day follow-up were not significantly different from those observed for 7-day follow-up (Figure S5 in Multimedia Appendix 1). However, level decreases associated with permanent billing code introduction were not significant for either outcome. Mean 14- and 30-day follow-up rates in the permanent billing code period were 21.75 (SD 3.00) and 32.32 (SD 3.91) per 100 first-contact visits.

#### Follow-Up Characteristics

In those with follow-up, the proportion conducted virtually was significantly larger in the temporary (1259/2428, 52%) and permanent (153/929, 16%) billing code periods than in the pre–virtual care period (86/11,502, 0.8%; Table S5 and Figure S6 in Multimedia Appendix 1). These proportions were approximately consistent across follow-up provider types in the temporary billing code period. In the permanent billing code period, 22% (96/437) of follow-up visits with psychiatrists were virtual vs 12% (57/454) of follow-up visits with general practitioners and pediatricians. Across all periods, follow-up was primarily delivered by general practitioners. Time to follow-up within 30 days increased over time. Before virtual care, most follow-ups (5821/11,502, 51%) took place within 7 days (median 7, IQR 3-15 days). In the temporary and permanent billing code periods, 48% (1160/2428) and 44% (406/929) of follow-ups took place within 7 days (median 8, IQR 4-15 days and 9, IQR 4-18 days, respectively).

#### Subgroup Analyses

In the pre–virtual care period, mean monthly rates of 7-day follow-up were lower among youth living in rural compared to urban areas (14.72, SD 7.41 vs 18.19, SD 3.02 per 100 first-contact visits) and in areas with lower compared to higher material resources (15.66, SD 3.94 vs 19.60, SD 4.18 per 100 first-contact visits; Figures S7 and S8 in Multimedia Appendix 1). Changes associated with the introduction of temporary and permanent billing codes were not significantly different between groups (Figures S9 and S10 in Multimedia Appendix 1). Lower mean rates in rural and lower material resource areas persisted throughout the temporary and permanent billing code periods.

### First-Contact Hospital Admissions

#### Visit Characteristics

This study included 17,701 first-contact hospital admissions for self-harm, a mood disorder, or psychosis: 13,603 (76.9%) in the pre–virtual care period (approximately 103 per month), 2876 (16.3%) in the temporary billing code period (approximately 96 per month), and 1222 (6.9%) in the permanent billing code period (approximately 81 per month; Table 2). First-contact hospital admissions rose from 2009 to 2017, then declined (Figure S11 in Multimedia Appendix 1). Differences in visit characteristics between the temporary and permanent billing code periods compared to the pre–virtual care period were observed in time of residence in Ontario (decreasingly shorter in each period) and primary care attachment (decreasingly lower in each period). Age, sex, diagnosis, rurality, and material resource quintile distributions were similar across periods.

**Table 2:** Characteristics of first-contact hospital admissions by period^a^.

Characteristics		Pre–virtual care period (March 1, 2009-February 29, 2020; n=13,603; approximately 103 per mo)	TBC^b^ period (June 1, 2020-November 31, 2022; n=2876; approximately 96 per mo)	SMD^c^—TBC vs pre–virtual care period	PBC^d^ period (December 1, 2022-February 29, 2024; n=1222; approximately 81 per mo)	SMD—PBC vs pre–virtual care period
**Age (y)**						
	Mean (SD)	18.46 (3.29)	18.46 (3.54)	<0.01	18.48 (3.57)	0.01
	Median (IQR)	18 (16-21)	19 (16-21)	0.01	18 (16-22)	0.01
**Age group (y), n (%)**						
	10-17	5683 (41.8)	1191 (41.4)	0.01	516 (42.2)	0.01
	18-24	7920 (58.2)	1685 (58.6)	0.01	706 (57.8)	0.01
**Sex, n (%)**						
	Female	7019 (51.6)	1588 (55.2)	0.07	653 (53.4)	0.04
	Male	6584 (48.4)	1288 (44.8)	0.07	569 (46.6)	0.04
**Diagnosis, n (%)**						
	Any self-harm	2933 (21.6)	599 (20.8)	0.02	256 (20.9)	0.01
	Mood disorder without self-harm	7782 (57.2)	1604 (55.8)	0.03	670 (54.8)	0.05
	Psychosis without self-harm	2888 (21.2)	673 (23.4)	0.05	296 (24.2)	0.07
**Immigration status, n (%)**						
	Resettled refugee or protected person and other immigrants^e^	471 (3.5)	137 (4.8)	0.07	66 (5.4)	0.09
	Sponsored family immigrants	392 (2.9)	87 (3)	0.01	42 (3.4)	0.03
	Economic immigrants	712 (5.2)	169 (5.9)	0.03	70 (5.7)	0.02
	Long-term residents	12,028 (88.4)	2483 (86.3)	0.06	1044 (85.4)	0.09
**Time of residence in Ontario (y), n (%)**						
	<5	713 (5.2)	233 (8.1)	*0.11* ^f^	132 (10.8)	*0.21*
	5-10	633 (4.7)	145 (5)	0.02	88 (7.2)	*0.11*
	>10	12,257 (90.1)	2498 (86.9)	*0.10*	1002 (82)	*0.24*
**Material resource quintile, n (%)**						
	1 (highest)	1969 (14.5)	389 (13.5)	0.03	158 (12.9)	0.04
	2	2206 (16.2)	516 (17.9)	0.05	172 (14.1)	0.06
	3	2371 (17.4)	528 (18.4)	0.02	240 (19.6)	0.06
	4	2721 (20)	503 (17.5)	0.06	225 (18.4)	0.04
	5 (lowest)	3728 (27.4)	801 (27.9)	0.01	360 (29.5)	0.05
	Missing	608 (4.5)	139 (4.8)	0.02	67 (5.5)	0.05
**Rurality, n (%)**						
	Rural	2032 (14.9)	393 (13.7)	0.04	153 (12.5)	0.07
	Urban	11,519 (84.7)	2467 (85.8)	0.03	1060 (86.7)	0.06
	Missing	52 (0.4)	16 (0.6)	0.03	9 (0.7)	0.05
**Primary care attachment^g^, n (%)**						
	Uncertainly attached, not receiving primary care services	1729 (12.7)	467 (16.2)	*0.10*	227 (18.6)	*0.16*
	Uncertainly attached, receiving primary care services	593 (4.4)	111 (3.9)	0.03	57 (4.7)	0.01
	Attached	11,108 (81.7)	2256 (78.4)	0.08	916 (75)	*0.16*
	Missing	173 (1.3)	42 (1.5)	0.02	22 (1.8)	0.04

^a^Table S1 in Multimedia Appendix 1 provides additional information on variable definitions. Parts of this material are based on data and/or information compiled and provided by Immigration, Refugees, and Citizenship Canada current to September 2024.

^b^TBC: temporary billing code.

^c^SMD: standardized mean difference.

^d^PBC: permanent billing code.

^e^“Protected person” refers to an individual granted refugee protection as a Convention refugee (individuals meeting the 1951 Geneva Convention definition of a refugee) or as a person in need of protection under Canadian law. Other immigrants are largely family members of refugees. They comprised <0.5% of the study population.

^f^Italicized values indicate meaningful differences between periods (standardized mean difference≥0.1).

^g^“Primary care attachment” refers to affiliation of a patient with a regular primary care provider (enrolled with a primary care physician, clinic, or center or having continuity of care with a given primary care physician). Uncertain attachment refers to patients who are unaffiliated with a primary care provider. Table S1 in Multimedia Appendix 1 provides additional information.

#### Receipt of 7-Day Mental Health Follow-Up

**Figure 3:**
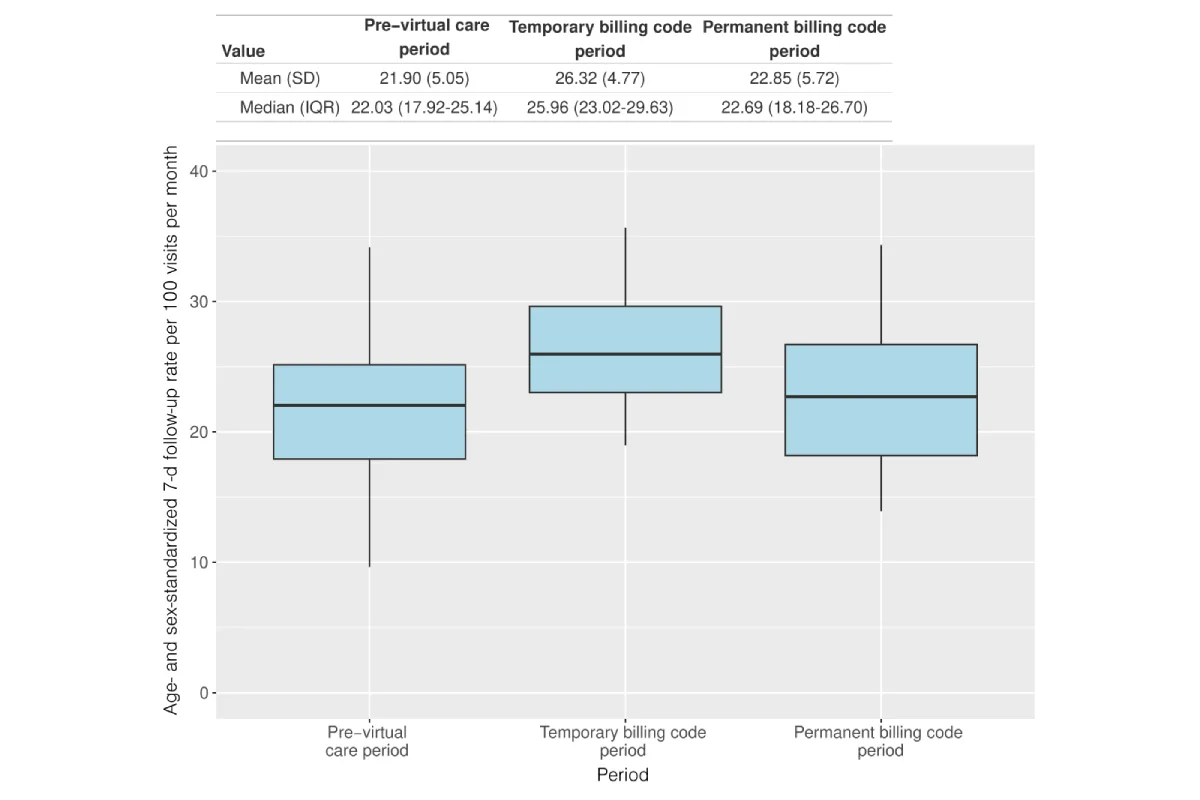
Distributions and average monthly rates of 7-day follow-up mental health care for first-contact hospital admissions by period.

**Figure 4:**
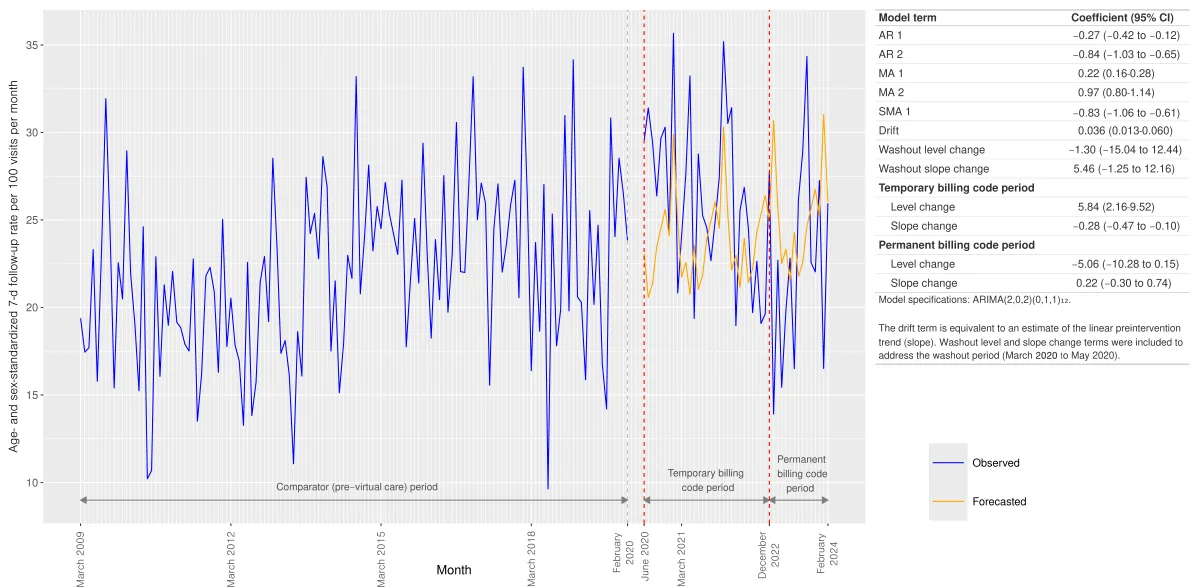
Time-series analysis results for monthly rates of 7-day mental health follow-up following first-contact hospital admissions for self-harm, mood disorders, or psychosis. AR 1: first-order autoregressive component; AR 2: second-order autoregressive component; ARIMA: autoregressive integrated moving average; MA 1: first-order moving average; MA 2: second-order moving average; SMA 1: first-order seasonal moving average.

In the pre–virtual care period, the mean standardized monthly rate of 7-day follow-up was 21.90 (SD 5.05) per 100 first-contact visits (Figure 3). Monthly follow-up rates rose slowly during this period at a rate of 0.036 follow-up visits per 100 per month (95% CI 0.013-0.060). Temporary billing code introduction was associated with an immediate, significant increase in 7-day follow-up (level change: 7.51, 95% CI 4.41-10.60), which then declined (slope change: −0.32, 95% CI −0.48 to −0.15; Figure 4). Permanent billing code introduction was not associated with significant changes in follow-up rates compared to the pre–virtual care period (level change: −4.01, 95% CI −8.19 to 0.18; slope change: −0.014, 95% CI −0.45 to 0.42). The mean standardized monthly rate of follow-up in the permanent billing code period was 21.75 (SD 3.00) per 100 first-contact visits. Sensitivity analyses and models with alternate differencing orders produced similar results (Tables S6 and S7 and Figure S12 in Multimedia Appendix 1).

#### Receipt of 14- and 30-Day Mental Health Follow-Up

In the pre–virtual care period, mean standardized monthly rates of 14- and 30-day follow-up were 34.53 (SD 5.21; 1.58 times higher than 7-day rates) and 49.28 (SD 6.42; 2.25 times higher than 7-day rates) per 100 first-contact visits (Figure S13 in Multimedia Appendix 1). Changes in 14- and 30-day follow-up were not significantly different from those observed for 7-day follow-up (Figure S14 in Multimedia Appendix 1). However, there was a significant level decrease in 14-day follow-up (−6.88, 95% CI −13.04 to −0.73) and slope increase in 30-day follow-up associated with permanent billing code introduction (0.64, 95% CI 0.0026-1.27). Mean 14- and 30-day follow-up rates in the permanent billing code period were 36.62 (SD 6.13) and 55.41 (SD 7.12) per 100 first-contact visits.

#### Follow-Up Characteristics

Among those with follow-up, the proportion conducted virtually was significantly higher in the temporary (788/1635, 48%) and permanent (81/676, 12%) billing code periods than before virtual care (84/6759, 1%; Table S8 and Figure S6 in Multimedia Appendix 1). These proportions were approximately consistent across follow-up provider types. Across all periods, the highest proportion of overall follow-ups was delivered by psychiatrists, and this increased over time. Most follow-ups took place beyond 7 days across all periods: 56% (3759/6759) before virtual care (median 9, IQR 4-17 days), 54% (885/1635) during the temporary billing code period (median 8, IQR 3-15 days), and 59% (399/676) in the permanent billing code period (median 11, IQR 4-18 days).

#### Subgroup Analyses

In the pre–virtual care period, mean rates of 7-day follow-up were lower in rural than in urban areas (15.04, SD 8.50 vs 23.01, SD 5.58 per 100 first-contact visits) and in areas with lower compared to higher material resources (20.47, SD 6.78 vs 23.25, SD 6.80 per 100 first-contact visits; Figures S15 and S16 in Multimedia Appendix 1). Excepting a significant difference in the slope change associated with permanent billing code introduction in rural compared to urban areas (−1.06, 95% CI −1.93 to −0.184 vs 0.44, 95% CI −0.179 to 1.06), changes in 7-day follow-up were not significantly different between groups (Figures S17 and S18 in Multimedia Appendix 1). However, while temporary billing code introduction was associated with significant level and slope changes in urban and higher–material resource areas, there were no significant changes in rural and lower–material resource areas. There was also a significant level decrease with permanent billing code introduction in areas with lower (−8.54, 95% CI −15.98 to −1.11) but not higher material resources. Lower mean rates in rural and lower–material resource areas persisted throughout the temporary and permanent billing code periods.

## Discussion

### Principal Findings

In our population-based repeated cross-sectional study of first-contact ED visits and hospital admissions for mood disorders, psychosis, and self-harm in Ontario youth and young adults, we found no association between the expansion of virtual care in Ontario and changes in rates of follow-up mental health care despite large increases in the proportion of follow-ups delivered virtually. These findings were supported by our sensitivity analyses. In subgroup analyses, we found no significant differences in the impact of virtual care expansion among youth and young adults living in rural compared to urban areas and areas with lower compared to higher material resources, excepting a significant decline in the rate of change of follow-up for rural but not urban hospital admissions with permanent billing code introduction.

Our analyses showed temporary increases in follow-up rates for both first-contact ED visits and hospital admissions associated with the introduction of temporary billing codes (June 2020), which then declined. These results are consistent with a population-based study of ED visits among Ontario youth that found increases in receipt of 60-day mental health follow-up for substance-related and all other ED visits from March 2020 to May 2021 compared to the 3 years prior (42% vs 33% and 14% vs 10%, respectively) [44]. A likely reason for the temporary increases observed was increased health system capacity for mental health care in the initial months of COVID-19 pandemic restrictions in mid-March 2020. In-person visits decreased drastically in these initial pandemic months [45], leaving physicians with temporarily increased capacity for services amenable to virtual delivery, such as mental health care. Indeed, approximately 70% of all child and adolescent outpatient mental health care in Ontario was delivered virtually from June 2020 to February 2021, an increase from less than 5% before the pandemic [21]. The decline in follow-up rates that followed could be explained by a decrease in health system capacity that resulted from a return to in-person appointments, normalization of care-seeking behaviors and disease epidemiology with the lifting of restrictions and school closures [46,47], the respiratory syncytial virus and influenza “twindemic” in the fall and winter of 2022 [48], and physician involvement in COVID-19 assessment and vaccination campaigns [49]. Involvement in COVID-19 assessment and vaccination was especially notable for general practitioners, who delivered the highest proportion of mental health follow-ups for first-contact ED visits (vs psychiatrists for hospital admissions). General practitioner capacity might have been additionally impacted by involvement in response to staffing shortages during acute care crises and delayed care [50], as well as the COVID-19 pandemic’s exacerbation of a preexisting family physician shortage in Ontario [51]. Another potential explanation for the temporary increase in follow-up is increased patient acuity (and, thus, indication for follow-up) due to ED avoidance in the early months of the pandemic or pandemic-related distress [52]. Mental health acute care use dropped in Ontario in the early months of the pandemic, with higher relative decreases for ED visits than for hospital admissions; however, these changes were concentrated during our washout period (March 2020-May 2020) [53]. Importantly, the changes in follow-up that we observed are unlikely to relate to dynamic responses to mental health need, reflecting the lack of a coordinated mental health care system in Ontario. Although early pandemic-related increases in patient acuity may have contributed to increased follow-up rates, there were likely also early pandemic-related increases in capacity for delivering mental health follow-up, suggesting that changes were due to the pandemic as opposed to system-level efforts.

Our results suggest a missed opportunity for virtual care to reduce inequities in access to follow-up care. For youth and young adults living in rural and lower–material resource areas, lower follow-up rates compared to their counterparts in urban and higher–material resource areas persisted across all study periods. Our findings are supported by an Ontario population-based study that found no meaningful changes in patient residence distance to medical specialists following virtual care expansion [54]. We observed wider gaps in follow-up for first-contact hospital admissions than for first-contact ED visits. This could be explained by higher indication for follow-up with a psychiatrist after hospital admission and less access to psychiatrists in rural and lower–material resource areas [55].

Importantly, our results also suggest a disparity in follow-up receipt among youth and young adults with first-contact ED visits and hospital admissions. Seven-day follow-up rates were low across all periods (14-19 per 100 first-contact ED visits and 22-26 per 100 first-contact hospital admissions). Comparatively, 7-day follow-up rates for all mental health–related hospital admissions among Ontario youth were approximately 32% to 38% from 2006 to 2014 [56]. Lower follow-up among first-contact youth would be consistent with prior findings of a strong association between recent connection to mental health services and receipt of mental health follow-up. Presence vs absence of recent (past-year or 6-month) connection to mental health care was found to be a major predictor of follow-up receipt among Ontario youth with substance use–related ED visits (adjusted odds ratio 6.86, 95% CI 6.57-7.17) [44]. Similar associations have been observed among multiple cohorts of privately and Medicaid-insured youth in the United States with mental health ED visits and hospitalizations [55,57,58]. First-contact youth are known to experience disparities in access to mental health care [1]. These disparities in access appear to persist in the postdischarge period for first-contact youth, and virtual care expansion seemingly did not help in addressing them.

With the introduction of permanent billing codes, the only significant change observed compared to the pre–virtual care period was a level decrease in follow-up rates for first-contact ED visits. The proportion of follow-ups delivered virtually was also significantly lower than in the temporary billing code period (81/676, 12% to 153/929, 16% vs 788/1635, 48% to 1259/2428, 52%). These results should be considered in the context of the preexisting downward trend in ED visit follow-up rates during the temporary billing code period, capacity changes affecting general practitioners, and the increase in the proportion of first-contact ED visits with youth not attached to a primary care provider (which doubled in the permanent billing code period compared to the pre–virtual care period). Changes in pandemic restrictions and changes to the virtual care billing structure might also have contributed to these findings. The permanent billing code structure distinguishes between virtual visits with and without a preexisting patient-physician relationship, with reduced compensation for the latter [32]. This would have affected a large proportion of our population; none would have had an ongoing relationship with a psychiatrist (unless previously seen for substance use visits), and at least 19% (227/1222) to 20% (573/2863) did not have an ongoing relationship with a primary care provider, with individuals in rural and lower material resource areas likely disproportionately represented. Overall, results from the permanent billing code period reflect that virtual care expansion failed to extend physician reach within Ontario’s universal health care system; preexisting barriers to care and system constraints persisted despite increased virtual delivery.

### Limitations

Our study had several limitations. First, the introduction of temporary billing codes was contemporaneous with the onset of the COVID-19 pandemic, making the impacts of the pandemic and virtual care inseparable in our analysis of this period. To help distinguish between pandemic- and virtual care–related impacts on mental health follow-up, we separately examined the later permanent billing code period (by which time pandemic restrictions were over and a steady state in virtual care use was expected). Second, ICES databases do not capture nonphysician community mental health care providers (eg, psychologists and social workers). As a result, some first-contact visits in our study might have been preceded by mental health care encounters with nonphysician providers, and we did not consider follow-up with nonphysician providers. However, we did not believe that prior receipt of nonphysician services would vary by period; there were no large systematic changes in funding for nonphysician care in Ontario during the study period. We also felt that a focus on physician follow-up was appropriate for first-contact self-harm, mood disorder, and psychosis ED visits and hospitalizations due to the high potential for individuals to be starting medications and guidelines recommending physician follow-up for these conditions. Third, there were reductions in primary care attachment and some demographic changes (increases in recent migrants, as well as in rural residence for first-contact ED visits) across our study periods that might have contributed to the reductions in follow-up rates observed. Fourth, we were not able to capture use of blended virtual and in-person postdischarge services or use of digital health tools. However, our focus was on the first follow-up visit and virtual care in Ontario’s universal health care system (telephone or video visits with a general practitioner or specialist).

### Conclusions

For youth whose first mental health contact for mood disorders, psychosis, or self-harm was through an ED visit or hospital admission, the expansion of virtual care was not associated with sustained changes in rates of mental health follow-up or improvements in preexisting inequities for those living in rural or lower material resource areas. Despite the clinical severity of the population and access to universal health insurance, follow-up rates were low across all periods. Although virtual follow-up increased, our findings suggest that it acted as an alternative to in-person care rather than a solution to system constraints and structural barriers to care. Future work should examine how to leverage the thus far missed opportunity for virtual care to extend physician reach in Ontario. Research into other potential benefits and harms of virtual care adoption is also needed, as well as strategies to ensure better follow-up among youth with first-contact visits.

## Appendix

Multimedia Appendix 1 Supplementary tables and figures.

## Abbreviations

ARIMA: autoregressive integrated moving average
ED: emergency department
PHIPA: Personal Health Information Protection Act

## Data Availability

The dataset from this study is held securely in coded form at ICES. While legal data sharing agreements between ICES and data providers (eg, health care organizations and government) prohibit ICES from making the dataset publicly available, access may be granted to those who meet prespecified criteria for confidential access, available [59] (email: das@ices.on.ca). The full dataset creation plan and underlying analytic code are available from the authors upon request, with the understanding that the computer programs may rely on coding templates or macros that are unique to ICES and are therefore either inaccessible or may require modification.
