# Supplementary material for: Virtual Care Expansion and Physician Follow-Up Among Youth With First Mental Health Contact Through a Hospital: Population-Based Repeated Cross-Sectional Study
Source: JMIR Ment Health. 2026 Sep 9;13:e94389. doi: 10.2196/94389 (PMC13601879; doi:10.2196/94389)
Supplement: Supplementary file 1 [file mental_v13i1e94389_app1.docx]

**Table S1: Population, exposure, outcome and variable definitions**

| **Variable** | **Source** | **Definition, categories** | **Time of measurement** |
| --- | --- | --- | --- |
| **Population** |  |  |  |
| First-contact ED visits or hospital admissions for mood disorders, psychosis, or self-harm among Ontario youth | NACRS, OHIP, CHC, DAD, OMHRS | Defined as unplanned ED visits or hospital admissions for mood disorders, psychosis, or self-harm among Ontario youth (ages 10-24) with no prior inpatient or outpatient mental health service contacts within the Ontario public healthcare system in the past 2 years.  Inclusion criteria:   1. ED visits (based on NACRS data): Discharged home between March 1, 2009 and February 29, 2024 following an unplanned ED visit for a mood disorder, psychosis, or self-harm.    a. Visits for mood disorders, psychosis, and self-harm were captured following existing mental health and addictions diagnostic groupings used in Ontario mental health system reporting, based on diagnostic field ICD-10-CA codes.    b. Mood disorders: F30, F31, F32, F33, F34, F38, F39, F53.0    c. Psychosis: F20 (excluding F20.4), F22, F23, F24, F25, F28, F29, F53.1    d. Self-harm: secondary diagnostic fields = X60-X84, Y10-Y19, Y28 when primary diagnostic field ne F06-F99 2. Hospital admissions (DAD, OMHRS): Discharged home between March 1, 2009 and February 29, 2024 following a hospital admission for a mood disorder, psychosis or self-harm, with admission via the ED following an unplanned ED visit (NACRS).    a. Admissions for mood disorders, psychosis, and self-harm were captured following existing mental health and addictions diagnostic groupings used in Ontario mental health system reporting, based on diagnostic field ICD-10-CA codes for DAD and ICD-9-CM codes (DSM-IV) for OMHRS.    b. Mood disorders: F30, F31, F32, F33, F34, F38, F39, F53.0; 296.x (all 296 codes), 300.4x, 301.13, 311.x    c. Psychosis: F20 (excluding F20.4), F22, F23, F24, F25, F28, F29, F53.1; 295.x (all 295 codes), 297.x (all 297 codes), 298.x (all 298 codes)    d. Self-harm: secondary diagnostic fields = X60-X84, Y10-Y19, Y28 when primary diagnostic field ne F06-F99   Exclusion criteria (same for ED visits and hospital admissions):   1. Non-Ontario resident 2. Invalid ICES key number (required for dataset linkage) 3. <10 or >24 years of age at discharge 4. Any inpatient or outpatient mental health service contact(s) for mental health conditions, excluding for substance use and addictions^1^, in the 2 years preceding the episode of care. This was based on any mental health service record in OHIP, CHC, DAD, and OMHRS:    a. OHIP: any prior 2 year mental health-related visits with a family physician, pediatrician, or psychiatrist    b. CHC: any prior 2 year mental health visits with a family physician, pediatrician, or psychiatrist    c. DAD/OMHRS: any prior 2 year mental health-related admissions to acute care or mental health hospitals    d. NACRS: any prior 2 year mental health-related ED visits in which patients were seen by a provider | N/A |
| **Time periods** | NACRS, DAD, OMHRS | First-contact emergency department visits and hospital admissions were divided into four time-based exposure, comparator and wash-out periods based on discharge date:   1. Pre-virtual care period: March 1, 2009 to February 29, 2020 2. Wash-out period: March 1 to May 30, 2020 (excluded) 3. Temporary billing code period: June 1, 2020 to November 30, 2022 4. Permanent billing code period: December 1, 2022 to February 29, 2024 | Discharge date (index) |
| **Outcome** |  |  |  |
| Age-sex standardized monthly rate of 7-, 14- and 30-day mental health follow-up | OHIP, CHC | Mental health follow-up was defined as presence vs absence of an outpatient mental health visit with a general practitioner, pediatrician, or psychiatrist within 7 (primary outcome), 14, or 30 days (secondary outcomes) of discharge. For ED visits, the outcome window was 1-30 days post-discharge; same-day (i.e., day 0) outpatient claims were excluded as there was high likelihood for them to not be representative of follow-up, but to instead correspond to claims related to the index ED visit. For hospital admissions, the outcome window was 0-30 days post-discharge; same-day outpatient claims were included in the follow-up definition as they should not overlap with inpatient claims. For each first-contact ED visit or hospital admission, a maximum of 1 follow-up visit (the first) was captured. Two databases were used to identify eligible outpatient mental health visits, beginning with pulling records within 7-30 days post-discharge:   1. OHIP: we applied an existing, validated algorithm^1^ developed at ICES for identification of mental health and addictions outpatient visits using OHIP claims, modified to exclude substance use and addictions related visits^2^. 2. CHC: we used information on visit provider type (“physician”, “pediatrician”, or “psychiatrist”) and ICD-10 codes for mental health conditions (F06-F09, F20-F54, F56-99, X60-X84, Y10-Y19) to identify eligible visits to CHCs.   We summed the number of 7-, 14-, and 30-day mental health follow-up visits in each index month (assigned based on discharge date) and calculated monthly follow-up rates as the number of mental health follow-up visits per 100 first-contact ED visits or hospital admissions per month. Rates were then age-sex standardized based on age-sex distributions of the ED visit and hospital admissions cohorts in 2023 (the last year of complete data); for subgroup analyses, standardization was based on subgroup-specific 2023 age-sex distributions. | ED: 1-30 days post-discharge  Hospital admission: 0-30 days post-discharge |
| Age-sex standardized monthly rate of follow-up for any indication, including planned ED visits (sensitivity analysis) | OHIP, CHC, NACRS | Defined as presence vs absence of an outpatient visit or planned emergency visit for any indication within 7 days post-discharge. For each first-contact ED visit or hospital admission, a maximum of 1 follow-up visit (the first) was captured. Three databases were used to identify eligible outpatient visits as follows:   1. OHIP: any OHIP claim within 7 days of discharge for an office-based or virtual visit 2. CHC: any visit to a CHC within 7 days of discharge 3. NACRS: any planned ED visit within 7 days of discharge   Calculation of rates and age-sex standardization was performed as described above. | ED: 1-30 days post-discharge  Hospital admission: 0-30 days post-discharge |
| **Subgroups** |  |  |  |
| Neighborhood material resources | ON-MARG Material Resources dimension; PCCF; RPDB | Categorized as lower (quintiles 4, 5, and missing) vs higher (quintiles 1, 2, and 3). Material resources quintiles were assigned at the census dissemination area level based on the residential postal code recorded in the RPDB at index; PCCF was used to convert postal codes into census dissemination areas (the smallest census geographic area, 400-700 people).  The ON-MARG material resources dimension is neighbourhood-level measure of socioeconomic status composed of area-level indicators such as proportion unemployment and without a highschool diploma. See eTable 1 for additional information. | Discharge date |
| Rurality | Rurality Index of Ontario | Categorized as rural (RIO score >/= 40) vs urban (RIO score <40).  The RIO is an Ontario-specific measure of rurality based on population size and density as well as travel time to the nearest basic and advanced referral centres. Scores are assigned by census subdivision area (municipalities or areas considered equivalent to municipalities) from 0 (most urban) to 100 (most rural) and are frequently dichotomized into rural vs. urban for research purposes using the above categorization. See eTable 1 for additional information. | Discharge date |
| **Baseline characteristics** |  |  |  |
| Age | OHIP RPBD (provincial health insurance registry) | Age in years at discharge date, based on date of birth in the RPDB. | Discharge date |
| Sex | OHIP RPDB (provincial health insurance registry) | Sex as indicated in the RPDB (currently limited to male or female). | Discharge date |
| Diagnosis | NACRS, DAD, OMHRS | Primary diagnosis associated with the first-contact ED visit or hospital admission. Categorized as mood disorder without self-harm, psychosis without self-harm, or self-harm. Mood disorder, psychosis, and self-harm definitions were based on existing mental health and addictions diagnostic groupings used in Ontario mental health system reporting (see population definition). | Discharge date |
| Immigration status | IRCC’s Permanent Resident Database | Categorized as economic immigrant, sponsored family, refugee/protected person, other immigration, and long-term resident. Economic immigrants, sponsored family, and refugee/protected person are representative of the main 3 immigration pathways in Canada; all other pathways were categorized as other immigration. Protected person refers to an individual granted refugee protection as a Convention refugee (individuals meeting the 1951 Geneva Convention definition of a refugee) or as a person in need of protection under Canadian law. Long-term residents were defined as those with no record in the IRCC’s Permanent Resident Database. | Discharge date |
| Time of residence in Ontario | IRCC’s Permanent Resident Database; RPDB | Categorized as <5 years, 5-10 years, and >10 years. Calculated as time in years from beginning of residence in Ontario (first of: OHIP eligibility date in RPDB or landing date in permanent resident database) to discharge date. | Start date of residence in Ontario to discharge date |
| Primary care attachment | PCPOP, CHC, ODB, ESTSOB | Categorized as attached, uncertainly attached receiving primary care services, uncertainly attached not receiving primary care services, and missing, using a validated primary care attachment algorithm developed at ICES^3^. Primary care attachment refers to affiliation of a patient with a regular primary care provider (enrolled with a primary care physician, clinic, or centre, or having continuity of care with a given primary care physician). Uncertain attachment with use of primary care services reflects patients who are unaffiliated with a primary care provider but are accessing primary care (1+ core primary care visits in the prior 2 years). Uncertain attachment without use of primary care services refers to patients who are unaffiliated with a primary care provider and are not accessing primary care (0 core primary care visits in the prior 2 years). Missing individuals were those who did not appear in the main database used in the algorithm (PCPOP). | Discharge date |
| **Follow-up characteristics** |  |  |  |
| Timing of follow-up post-discharge | NACRS, OHIP, CHC, DAD, OMHRS | Time in days between discharge date and follow-up date. | Discharge date to follow-up date |
| Follow-up modality | OHIP | Categorized as virtual (video or phone) vs. in-person (office, home, or long-term care/complex care home). Defined using ICES variable for OHIP claim location, which is based on billing codes. Visit modality is not currently captured in the CHC database; follow-up visits at CHCs (<5% of follow-up) were all categorized as in-person. | Follow-up date |
| Follow-up provider | OHIP, CHC | Categorized as general practitioner, pediatrician, or psychiatrist. Defined as the specialty of the physician who provided follow-up based on provincial health insurance plan billing and CHC records. | Follow-up date |

CHC = Community Health Centre; DAD = Discharge Abstract Database; ED = emergency department; ESTSOB = Estimated Schedule of Benefits; IRCC = Immigration, Refugees, and Citizenship Canada, NACRS = National Ambulatory Care Reporting System; ODB = Ontario Drug Benefit Claims; OHIP = Ontario Health Insurance Plan; OMHRS = Ontario Mental Health Reporting System; PCPOP = Primary Care Population; RPDB = Registered Persons Database

See Table S2 for additional information on databases.

^1^Steele LS, Glazier RH, Lin E, Evans M. Using Administrative Data to Measure Ambulatory Mental Health Service Provision in Primary Care. Medical Care. 2004;42(10)

^2^Substance use and addictions visits were not included in our definitions of prior outpatient and inpatient mental health service use or in our definition of mental health follow-up. Our rationale for this was that substance use and addictions visits in youth are 1) more likely to represent one-time services (e.g., treatment for alcohol poisoning) and are thus less likely to influence future access to mental health services.

^3^Jaakkimainen L, Bayoumi I, Glazier RH, et al. Development and validation of an algorithm using health administrative data to define patient attachment to primary care providers. J Health Organ Manag. 2021;ahead-of-print(ahead-of-print):733-743. doi:10.1108/JHOM-05-2020-0171

**Table S2: Database descriptions**

| **Database** | **Source** | **Description** |
| --- | --- | --- |
| OHIP Registered Persons Database (RPDB) (provincial healthcare registry) | Canadian Institute for Health Information (CIHI), Ontario Ministry of Health | Basic demographic information (postal code, age, sex, date of birth, and date of death) for all persons with an Ontario health insurance number, as well as time period of health insurance eligibility. |
| Canadian Institute for Health Information – Discharge Abstract (DAD) Database | Canadian Institute for Health Information (CIHI), Ontario Ministry of Health | Clinical, administrative, and demographic information on admissions to acute care hospitals in Ontario. Consecutive hospital admissions are linked at ICES to form episodes of care (e.g., if an individual is transferred from one hospital to another). |
| National Ambulatory Care Reporting System (NACRS) | Canadian Institute for Health Information (CIHI), Ontario Ministry of Health | Clinical, administrative, and demographic information on visits to hospital- and community-based ambulatory care centres (emergency departments, etc.) in Ontario. At ICES, linkage with other data sources (e.g., the DAD) enables identification of transitions from ambulatory to inpatient care and formation of episodes of care. |
| Ontario Health Insurance Plan Claims Database (OHIP) | Ontario Ministry of Health | Clinical and administrative information on inpatient and outpatient services provided by physicians and covered by OHIP. Service records enable identification of the type of service, the location of service, the provider, and the provider’s specialization, as well as the main reason for the service, captured using diagnostic codes. |
| Community Health Centre Database (CHC) | Community Health Centres | Clinical, administrative, and demographic information on primary care services provided at community health centres (CHCs) in Ontario. |
| Ontario Mental Health Reporting System (OMHRS) | Canadian Institute for Health Information (CIHI), Ontario Ministry of Health | Administrative, clinical, and demographic information on all adults and some youth receiving inpatient mental health services in Ontario. Data is collected at hospitals using the Resident Assessment Instrument – Mental Health and submitted to CIHI. Assessments are completed at admission and discharge, as well as every 3 months for individuals with extended admissions and when there are significant, unexpected changes in clinical status. At ICES, consecutive OMHRS as well as DAD records for a given patient can be linked to form episodes of care. |
| Immigration, Refugees and Citizenship Canada’s Permanent Resident Database (CIC) | Immigration, Refugees and Citizenship Canada (IRCC) | Demographic information on permanent residents who originally landed in Ontario, collected during the immigration application process. The overall linkage rate between the IRCC database and the Ontario healthcare registry (RPDB) is estimated to be 86.4%.^1^ |
| Primary care population (PCPOP) | Canadian Institute for Health Information (CIHI), Ontario Health, Ontario Ministry of Health | ICES-derived, population-level dataset containing data on Ontario residents’ primary care roster status (formal registration with a primary care physician, team, or centre) and additional administrative information pertaining to primary care. For inclusion in the dataset, an individual must be alive, a resident of Ontario, have had contact with the healthcare system within the past 7-9 years, and be eligible for the provincial health insurance plan (OHIP). |
| Ontario Marginalization Index (ON-MARG) Material Resources dimension | Ontario Community Health Profiles Partnership | Neighbourhood-level measure of marginalization in Ontario, based on Canadian census data. It is updated every 5 years, following the Canadian census schedule. We used ON-MARG 2011, 2016, and 2021 in this study, selecting the most relevant version for each year of our dataset (e.g., ON-MARG 2016 was used for visits/admissions in 2014-2018). The full ON-MARG index contains 4 dimensions: material resources (formerly “residential instability”), households and dwellings (formerly “material deprivation”), age and labour force (formerly “dependency”), and racialized and newcomer populations (formerly “ethnic concentration”). The material resources dimension, which reflects socioeconomic status, was used in this study. It includes the following area-level, proportion-based indicators (with some variation based on ON-MARG year): aged 25-64 without a high-school diploma, families who are lone parent families, income from government transfer payments, unemployment, individuals considered low-income, households living in dwellings in need of repair. The full index and its dimensions are reported by census dissemination area (the smallest census geographic area, 400-700 individuals) or higher-level census geographies (e.g., census tracts) in quintiles ranging from least to most deprived. |
| Postal Code Conversion File (PCCF)+ | Statistics Canada | Enables conversion of postal code to standard geographic areas from the census. It was used to identify census dissemination areas for use with ON-MARG. |
| Rurality Index of Ontario | Ontario Medical Association | Neighbourhood-level measure of rurality in Ontario, ranging from 0 (most urban) to 100 (most rural). This measure broadens traditional, population size- and density-based measures of rurality to also include healthcare access specific measures of rurality (travel time to nearest basic and advanced referral centres). It consists of three differentially-weighted measures which are summed and transformed to a 0-100 score: 1) community population and population density (points are given linearly to communities with populations <45,000, with additional points for low population density) (assigned a weight of 28.6%), 2) travel time to nearest basic referral centre (assigned a weight of 47.6%), and 3) travel time to nearest advanced referral centre (assigned a weight of 23.8%). Measures are relative to provincial medians. Calculation and reporting is by Canadian census subdivision area (municipalities or areas considered equivalent to municipalities). |
| Ontario Drug Benefit Claims (ODB) | IQVIA Solutions Canada Inc., Ontario Ministry of Health | Administrative information on claims made for drugs covered by the Ontario Drug Benefit program. Required for the primary care attachment algorithm (see eTable 2). |
| Estimated Schedule of Benefits (ESTSOB) | Ontario Ministry of Health | Prices associated with each OHIP fee code or suffix. Required for primary care attachment algorithm (see eTable 2). |

^1^Chiu M, Lebenbaum M, Lam K, Chong N, Azimaee M, Iron K, Manuel D, Guttmann A. *BMC Med Inform Decis Mak*. 2016; 16(1):135. Epub 2016 Oct 21

**Table S3: Time series analysis results for monthly 7-day mental health follow-up rates for first-contact emergency department visits, estimated using a segmented linear regression model**

**Table S4: Time series analysis results for monthly 7-day mental health follow-up rates for first-contact emergency department visits, estimated using ARIMA models with alternate differencing orders (first order difference and no differencing)**

The level change associated with permanent billing code introduction was not significant with a first order difference or no differencing but was significant with the differencing order used for primary analyses (second order difference).

**Table S5: Mental health follow-up characteristics in the temporary and permanent billing code periods compared to the pre-virtual care period, first-contact emergency department visits**

| **Characteristic** | **Value** | **Pre-virtual care period (March 1, 2009 to February 29, 2020)** | **Temporary billing code period (June 1, 2020 to November 31, 2022)** | **SD (temporary billing code vs pre-virtual care period)** | **Permanent billing code period (December 1, 2022 to February 29, 2024)** | **SD (permanent billing code vs pre-virtual care period)** |
| --- | --- | --- | --- | --- | --- | --- |
|  |  | N=33,076 | N=6,269 |  | N=2,863 |  |
| Timing of follow-up post-discharge (count in days) | Mean ± standard deviation | 9.97 ± 8.08 | 10.22 ± 7.97 | 0.03 | 11.12 ± 8.28 | **0.14** |
|  | Median (IQR) | 7 (3-15) | 8 (4-15) | 0.05 | 9 (4-18) | **0.15** |
| Timing of follow-up post-discharge | 1-7 days | 5,821 (17.6%) | 1,160 (18.5%) | 0.02 | 406 (14.2%) | 0.09 |
|  | 8-14 days | 2,618 (7.9%) | 609 (9.7%) | 0.06 | 220 (7.7%) | <0.01 |
|  | 15-30 days | 3,063 (9.3%) | 659 (10.5%) | 0.04 | 303 (10.6%) | 0.04 |
|  | None | 21,574 (65.2%) | 3,841 (61.3%) | 0.08 | 1,934 (67.6%) | 0.05 |
| Follow-up modality | Virtual | 86 (0.3%) | 1,259 (20.1%) | **0.69** | 153 (5.3%) | **0.31** |
|  | In-person | 11,416 (34.5%) | 1,169 (18.6%) | **0.37** | 776 (27.1%) | **0.16** |
|  | None | 21,574 (65.2%) | 3,841 (61.3%) | 0.08 | 1,934 (67.6%) | 0.05 |
| Follow-up provider | General practitioner | 6,000 (18.1%) | 1,211 (19.3%) | 0.03 | 454 (15.8%) | 0.06 |
|  | Pediatrician | 363 (1.1%) | 83 (1.3%) | 0.02 | 38 (1.3%) | 0.02 |
|  | Psychiatrist | 5,139 (15.5%) | 1,134 (18.1%) | 0.07 | 437 (15.3%) | 0.01 |
|  | None | 21,574 (65.2%) | 3,841 (61.3%) | 0.08 | 1,934 (67.6%) | 0.05 |
| Follow-up provider, virtual follow-up | General practitioner or pediatrician^1^ | 8 (0.02%) | 663 (10.0%) | **0.48** | 57 (1.8%) | **0.20** |
|  | Psychiatrist | 78 (0.2%) | 596 (9.5%) | **0.44** | 96 (3.4%) | **0.24** |
|  | In-person | 11,416 (34.5%) | 1,169 (18.6%) | **0.37** | 776 (27.1%) | **0.16** |
|  | None | 21,574 (65.2%) | 3,841 (61.3%) | 0.08 | 1,934 (67.6%) | 0.05 |

IQR = interquartile range; SD = standardized difference

^1^Pediatrician was merged with general practitioner due to small cell counts for pediatricians (<6) in some columns.

**Table S6: Time series analysis results for monthly 7-day mental health follow-up rates for first-contact hospital admissions, estimated using a segmented linear regression model**

The permanent billing code period level decrease was significant with use of this model and not in the primary analysis.

**Table S7: Time series analysis results for monthly 7-day mental health follow-up rates for first-contact hospital admissions, estimated using ARIMA models with alternate differencing orders (first order difference and no differencing)**

**Table S8: Mental health follow-up characteristics in the temporary and permanent billing code periods compared to the pre-virtual care period, first-contact hospital admissions**

| **Characteristic** | **Value** | **Pre-virtual care period (March 1, 2009 to February 29, 2020)** | **Temporary billing code period (June 1, 2020 to November 31, 2022)** | **SD (temporary billing code vs pre-virtual care period)** | **Permanent billing code period (December 1, 2022 to February 29, 2024)** | **SD (permanent billing code vs pre-virtual care period)** |
| --- | --- | --- | --- | --- | --- | --- |
|  |  | N=13,603 | N=2,876 |  | N=1,222 |  |
| Timing of follow-up post-discharge (count in days) | Mean ± standard deviation | 10.72 ± 8.54 | 10.29 ± 8.55 | 0.05 | 11.52 ± 8.92 | 0.09 |
|  | Median (IQR) | 9 (4-17) | 8 (3-15) | 0.06 | 11 (4-18) | 0.08 |
| Timing of follow-up post-discharge | 0-7 days | 3,000 (22.1%) | 750 (26.1%) | 0.09 | 277 (22.7%) | 0.01 |
|  | 8-14 days | 1,703 (12.5%) | 418 (14.5%) | 0.06 | 169 (13.8%) | 0.04 |
|  | 15-30 days | 2,056 (15.1%) | 467 (16.2%) | 0.03 | 230 (18.8%) | **0.10** |
|  | None | 6,844 (50.3%) | 1,241 (43.2%) | **0.14** | 546 (44.7%) | **0.11** |
| Follow-up modality | Virtual | 84 (0.6%) | 788 (27.4%) | **0.84** | 81 (6.6%) | **0.33** |
|  | In-person | 6,675 (49.1%) | 847 (29.4%) | **0.41** | 595 (48.7%) | 0.01 |
|  | None | 6,844 (50.3%) | 1,241 (43.2%) | **0.14** | 546 (44.7%) | **0.11** |
| Follow-up provider | General practitioner | 2,901 (21.3%) | 611 (21.2%) | <0.01 | 223 (18.2%) | 0.08 |
|  | Pediatrician | 200 (1.5%) | 43 (1.5%) | <0.01 | 13 (1.1%) | 0.04 |
|  | Psychiatrist | 3,658 (26.9%) | 981 (34.1%) | **0.16** | 440 (36.0%) | **0.20** |
|  | None | 6,844 (50.3%) | 1,241 (43.2%) | **0.14** | 546 (44.7%) | **0.11** |
| Follow-up provider, virtual follow-up only | General practitioner or pediatrician^1^ | 1-6 (0.007-0.04%)^2^ | 333 (11.6%) | **0.50-0.51**^2^ | 26 (2.1%) | **0.20-0.21**^2^ |
|  | Psychiatrist | 78-83 (0.57-0.61%)^2^ | 455 (15.8%) | **0.57-0.58**^2^ | 55 (4.5%) | **0.24-0.26**^2^ |
|  | In-person | 6,675 (49.1%) | 847 (29.4%) | **0.41** | 595 (48.7%) | 0.01 |
|  | None | 6,844 (50.3%) | 1,241 (43.2%) | **0.14** | 546 (44.7%) | **0.11** |

IQR = interquartile range; SD = standardized difference

^1^Pediatrician was merged with general practitioner due to small cell counts for pediatricians (<6) in some columns.

^2^Reported as a range due to risk of reidentification with small cell counts (<6).

**Figure S1: Cohort creation diagram**

**National Ambulatory Care Reporting System, Discharge Abstract Database, Ontario Mental Health Reporting System**

Emergency department visits and hospital admissions for self-harm, psychosis, and mood disorders resulting in discharge home from Mar 1, 2009 to Feb 29, 2024

n = 1,122,027

First-contact hospital admissions for self-harm, psychosis, and mood disorders among Ontario residents aged 10-24 resulting in discharge home from Mar 1, 2009 to Feb 29, 2024, excluding Mar-May 2020 wash-out period

n = 17,701

First-contact emergency department visits for self-harm, psychosis, and mood disorders among Ontario residents aged 10-24 resulting in discharge home from Mar 1, 2009 to Feb 29, 2024, excluding Mar-May 2020 wash-out period

n = 42,208

Link to Ontario Health Care Registry (Registered Persons Database)

Link to outpatient and inpatient service records (Ontario Health Insurance Plan, Community Health Centre, National Ambulatory Care Reporting System, Discharge Abstract Database, Ontario Mental Health Reporting System)

Outpatient or inpatient mental health service contact(s) in the two years preceding the episode of care n = 275,046

Discharge during wash-out period (Mar 1 to May 30, 2020) n = 213

Non-Ontario resident n = 3,079

Age <10 or >24 at discharge n = 783,349

Discharge during wash-out period (Mar 1 to May 30, 2020) n = 431

First-contact emergency department visits and hospital admissions for self-harm, psychosis, and mood disorders among Ontario residents aged 10-24 resulting in discharge home from Mar 1, 2009 to Feb 29, 2024

n = 60,553 (non-admitted: n = 42,639; admitted n = 17,914)

**Figure S2: Total monthly first-contact emergency department visits for self-harm, psychosis, and mood disorders among youth 10-24 years old in Ontario, March 2009 to February 2024**

**Figure S3: Time series analysis results for monthly rates of 7-day follow-up for any indication (including planned emergency department visits) following first-contact emergency department visits for self-harm, mood disorders, or psychosis**

**Figure S4: Distributions and average monthly rates of 14-day and 30-day mental health follow-up for first-contact emergency department visits, by time period**

**Figure S5: Time series analysis results for monthly rates of 14- and 30-day mental health follow-up following first-contact emergency department visits for self-harm, mood disorders, or psychosis**

**A. 14-day mental health follow-up**

**B. 30-day mental health follow-up**

**Figure S6: Overall rates of virtual, in-person, and no follow-up within 30 days in each time period**

B. First-contact hospital admissions

Proportion of total first-contact visits

| Time period | Virtual follow-up | In-person follow-up | No follow-up |
| --- | --- | --- | --- |
| Pre-virtual care (N=13,603) | 84 | 6675 | 6844 |
| TBC period (N=2,876) | 788 | 847 | 1241 |
| PBC period (N=1,222) | 81 | 595 | 546 |

A. First-contact emergency department visits

Proportion of total first-contact visits

| Time period | Virtual follow-up | In-person follow-up | No follow-up |
| --- | --- | --- | --- |
| Pre-virtual care (N=33,076) | 8600% | 1141600% | 2157400% |
| TBC period (N=6,629) | 125900% | 116900% | 384100% |
| PBC period (N=2,863) | 15300% | 77600% | 193400% |

PBC = permanent billing code, TBC = temporary billing code

**Figure S7: Distributions and average monthly rates of 7-day mental health follow-up for first-contact emergency department visits by time period, by residence in rural vs. urban areas**

**Figure S8: Distributions and average monthly rates of 7-day mental health follow-up for first-contact emergency department visits by time period, by residence in lower vs. higher material resources areas**

**Figure S9: Time series analysis results for monthly rates of 7-day mental health follow-up following first-contact emergency department visits for self-harm, mood disorders or psychosis, by residence in rural vs. urban areas**

**A. Rural areas**

**B. Urban areas**

152 months of 177 in the rural group had outcome counts <10; standardized rates for these months may be unreliable.

**Figure S10: Time series analysis results for monthly rates of 7-day mental health follow-up following first-contact emergency department visits for self-harm, mood disorders or psychosis, by residence in lower vs. higher material resources areas**

 **A. Lower material resources areas**

**B. Higher material resources areas**

13 months of 177 in the lower material resources group and 10 months of 177 in the higher material resources group had outcome counts <10; standardized rates for these months may be unreliable.

**Figure S11: Total monthly first-contact hospital admissions for self-harm, mood disorders, and psychosis among youth 10-24 years old in Ontario, March 2009 to February 2024**

**Figure S12: Time series analysis results for monthly rates of 7-day follow-up for any indication (including planned emergency department visits) following first-contact hospital admissions for self-harm, mood disorders, or psychosis**

The temporary billing code slope change was not significant in this sensitivity analysis but was significant in the primary analysis.

**Figure S13: Distributions and average monthly rates of 14-day and 30-day mental health follow-up for first-contact hospital admissions, by time period**

**Figure S14: Time series analysis results for monthly rates of 14- and 30-day mental health follow-up following first-contact hospital admissions for self-harm, mood disorders, or psychosis**

**A. 14-day mental health follow-up**

**B. 30-day mental health follow-up**

**Figure S15: Distributions and average monthly rates of 7-day mental health follow-up for first-contact hospital admissions by time period, by residence in rural vs. urban areas**

**Figure S16: Distributions and average monthly rates of 7-day mental health follow-up for first-contact hospital admissions by time period, by residence in lower vs. higher material resources areas**

**Figure S17: Time series analysis results for monthly rates of 7-day mental health follow-up following first-contact hospital admissions for self-harm, mood disorders or psychosis, by residence in rural vs. urban areas**

 **A. Rural areas**

**B. Urban areas**

All 177 months in the rural group and 7 months of 177 in the urban group had outcome counts <10; standardized rates for these months may be unreliable.

**Figure S18: Time series analysis results for monthly rates of 7-day mental health follow-up following first-contact hospital admissions for self-harm, mood disorders or psychosis, by residence in lower vs. higher material resources areas**

 **A. Lower material resources areas**

**B. Higher material resources areas**

65 months of 177 in the lower material resources group and 56 months of 177 in the higher material resources group had outcome counts <10; standardized rates for these months may be unreliable.
